# COVID 19 outbreak: impact of the quarantine-induced stress on cardiovascular disease risk burden

**DOI:** 10.2217/fca-2020-0055

**Published:** 2020-04-30

**Authors:** Anna Vittoria Mattioli, Milena Nasi, Camilla Cocchi, Alberto Farinetti

**Affiliations:** 1 Surgical, Medical & Dental Department of Morphological Sciences related to Transplant, Oncology & Regenerative Medicine, University of Modena & Reggio Emilia, Italy; 2 Istituto Nazionale per le Ricerche Cardiovascolari, University of Modena, Italy

**Keywords:** COVID-19, lifestyle, physical activity, quarantine, stress

The novel Coronavirus, CoV-19/SARS-CoV-2 is causing a global pandemic with a high number of deaths [1,2]. Although pandemic preparation plans have been developed, little attention has been paid to the cardiovascular burden of such an event [2].

Cardiovascular risk factors are strongly influence by quarantine, an effective measure that minimizes the impact of infectious disease outbreaks [3]. These restrictions will strongly influence life-styles leading to an increased burden of cardiovascular disease [4].

Previous research has revealed a profound and wide range of psychosocial impacts on people at the individual, community and international levels during outbreaks of infection [5]. Psycological distress is an important side effect of quarantine [6]. Mass-quarantine, self-quarantine and isolation are associated with depression, anger, and chronic stress. The stressor factors suggested included longer quarantine duration, frustration, boredom, inadequate supplies, inadequate information, financial loss and stigma. In addition, during outbreak, people are fearful of falling sick or dying themselves. These negative feelings are associated with systemic inflammation and endothelial dysfunction as well as tendency to adopt an unhealthy lifestyle [7]. Both acute and sub-acute stress activates the adrenergic system and increase inflammatory response and endothelial dysfunction leading to an increase in atherosclerotic plaques [5,8,9]. Some type of stress preceded a substantial percentage of myocardial infarctions. This was reported after the occurrence of earthquakes [10]. The reason has been associated with changes in neurohormonal, hemodynamic and coagulation systems that cause rupture of a vulnerable atherosclerotic plaque, platelet activation and coronary vasoconstriction. Events like these may prompt the emergence of collective stress among the population. The same occur during pandemics as well.

The sympathetic system activation influences cardiovascular system in several ways: increase heart rate and ventricular contractility, increase blood pressure, increase systemic and coronary resistance, promote thrombus formation and increase the risk of arrhythmias.

The hypothalamic-pituitary-adrenocortical axis releases plasmatic cortisol that increases blood pressure and plasma glucose levels. Moreover, cortisol alters the platelet function and the systemic inflammatory response. Corticotrophin-releasing hormone produced by hypothalamic-pituitary-adrenocortical axis increases the inflammatory response, macrophage activation, adhesion of monocyte to endothelial cell and endothelin-1 release.

Previous studies found that mental stress induced paradoxical vasoconstriction at the level of coronary artery stenosis and the degree of vasoconstriction is correlated with the degree of atherosclerosis [11].

In addition acute or chronic stress negatively influenced nutritional behaviors such as alcohol consumption, smoking and diet [12,13]. Some individuals respond to stress by eating more and selected foods high in sugar and fat [12,13]. This emotional eating may contribute to excess energy intakes and weight gain [12–14]. Torres and coworkers identified that people cope with stress by eating and drinking in an attempt to feel better (‘stress-related eating’) [14]. These stress driven eaters and drinkers were more likely to consume unhealthy foods and alcohol. Moreover, the lack of emotional support from friends and relatives increased stress driven eating and drinking behaviors [14].

Quarantine is associated with a diet poor in fresh fruit and vegetables, however it is known that higher vegetable intake is correlated with lower anxiety and fear severity. A previous study found that higher nonrefined grain consumption is significantly related to lower depression and anxiety compared with controls and these relationships persisted after adjustment for other food groups [15]. The Mediterranean diet is a healthy diet rich in vegetables, fruits and nonrefined grains. It is interesting that higher consumption of nonrefined grains, vegetables, fruits, potatoes, fish and olive oil were inversely related to depression or anxiety severity, while higher consumption of poultry and high fat dairy products was positively associated with higher anxiety symptoms. During the quarantine, changes in the diet contribute to increasing the stress and depression associated with the isolation.

In addition, emotional eating and reduction of physical activity lead to obesity and metabolic syndrome, both risk factors has a pivotal role in cardiovascular risk. Obesity is also associated with an increase risk of Type 2 diabetes [8,9].

Technologies could be useful; a high number of health and nutrition applications are available on Google play and the Apple app store. App programs may help the self-control of diet and to maintain personal ideal weight. The social support improves the use of these tools for adopting a healthy lifestyle.

Physical activity as well as relaxing activity could be useful in reducing stress during quarantine. However, the limitation imposed by government restrictions on outdoor activities affects the vast majority of physical activities. The main consequence of quarantine has been the reduction of physical activity. Physical activity motivation is strongly related to social aspects, such as indoor gym groups and team competitions. These activities were blocked and it is presumable that people will reduce exercise. In order to reduce the negative effects of quarantine on health the WHO has just released guidance to “*Stay physically active during self-quarantine*” [16]. These indications are intended for people in self-quarantine without any symptoms or diagnosis of acute respiratory illness and contained practical advice on how to stay active and reduce sedentary behavior while at home. To get these objectives, new technologies and internet could be useful; on-line exercise classes and video- or app-guided aerobics training at home can be a simple and economic tool for performing physical activity. Therefore, to not change the healthy lifestyle habits and to maintain an active behavior at home is very important for the health of the overall population but, especially, for subjects with cardiovascular and metabolic risk factors and for older people [8]. There are several options for exercising and training at home; aerobic exercises like walking inside the house and dancing can be easily done. Resistance training can be obtained by going up and down a step or the home stairs, sitting and getting up from a chair and transporting items with light and moderate weights. Exercise and physical activity play a pivotal role in prevention of cardiovascular disease [17]. Limited physical activity, sedentary behavior and sitting time are associated with increased risk of cardiovascular disease and with several metabolic and mental effects that also would contribute to increase the cardiovascular risk [8,9,17]. There are several mechanisms through which exercise training reduces chronic inflammation, including improvement of endothelial function and the capacity to regenerate endothelium after injury [9].

WHO suggest that meditation and deep breaths can help to remain calm and reduce stress.

A review analyzed the relationship between yogic practice and decline in anxiety and stress and concluded that scientific studies do not report significant reduction. However, due to the self-reported beneficial outcomes for the use of yoga as an intervention for stress and anxiety, yoga may be considered as a possible adjunctive therapy for those experiencing stress during quarantine. The word yoga, meaning ‘union’, is a mind-body-spirit practice that can include meditation, breath awareness, asanas or postures and relaxation. It is thought to alter nervous system regulation, physiology, psychological well-being and physical fitness. Due to the difficulties in performing exercise in the right way, the practice could be useful in people familiar with this technique [18].

## Conclusion

There is a strong relationship between cardiovascular system and SARS-CoV-2. The virus could affect cardiovascular system by several mechanisms: direct myocardial injury, systemic inflammation, altered myocardial demand-supply ratio, plaque rupture and coronary thrombosis, adverse effects of various therapies (i.e., prolonged QT interval) and electrolyte imbalances [19].

Patients with previous cardiovascular disease (CVD) appear to be more inclined to develop COVID-19 and have more severe clinical disease with worse outcome [19]. Previous cardiovascular disease, are associated with threefold greater risk of severe COVID 19 disease requiring in many cases intensive cares. Similarly, several cardiovascular risk factors (i.e., diabetes and hypertension) adversely affect prognosis of these patients.

We cannot exclude that quarantine stress could lead to Takotsubo Syndrome due to the sympathetic nervous system hyper-activation. A case of myocarditis, as a possible late phenomenon of the COVID 19 respiratory infection, has recently been reported from a group of Brescia hospital [20].

And finally the quarantine for containing the Covid 19 outbreaks affect cardiovascular risk factors leading to an increase of cardiovascular risk burden.
